# NMR Observation of Mobile Protons in Proton-Implanted ZnO Nanorods

**DOI:** 10.1038/srep23378

**Published:** 2016-03-18

**Authors:** Jun Kue Park, Hyeok-Jung Kwon, Cheol Eui Lee

**Affiliations:** 1 Korea Multi-purpose Accelerator Complex, Korea Atomic Energy Research Institute, Gyeongju 38180, Korea; 2 Korea University of Science and Technology, Daejon 34113, Korea; 3 Department of Physics, Korea University, Seoul 02841, Korea

## Abstract

The diffusion properties of H^+^ in ZnO nanorods are investigated before and after 20 MeV proton beam irradiation by using ^1^H nuclear magnetic resonance (NMR) spectroscopy. Herein, we unambiguously observe that the implanted protons occupy thermally unstable site of ZnO, giving rise to a narrow NMR line at 4.1 ppm. The activation barrier of the implanted protons was found to be 0.46 eV by means of the rotating-frame spin-lattice relaxation measurements, apparently being interstitial hydrogens. High-energy beam irradiation also leads to correlated jump diffusion of the surface hydroxyl group of multiple lines at ~1 ppm, implying the presence of structural disorder at the ZnO surface.

Electronic and diffusion properties of hydrogen in ZnO have been extensively studied, since efficient defect engineering is essential to fabricate electronic, ferroelectric, and optical devices^1^,^2^,^3^,^4^. Furthermore, diffusion properties of H donors may help in understanding the mechanism responsible for ferromagnetic order triggered by proton implantation^5^. The electronic characteristics on ZnO can be modified by proton irradiation, as the physical origin is attributed to the hydrogen shallow donors^6^,^7^,^8^,^9^. Recently, it has also been reported that radiation-induced defects cause the threshold voltage shift and the electrical conductance modulation, making them potentially applicable in nanoelectronic devices^7^,^10^. Although previous works addressed effects of proton implantation on electrical properties, it is still far from being well understood from a microscopic point of view. Here, we observe atomic-scale features on protons and their diffusion properties in proton-implanted ZnO nanorods by using proton NMR spectroscopy.

Extensive works have been made to investigate the site and stability of interstitial H (H_*i*_) in ZnO by employing infrared and secondary ion mass spectroscopy^11^,^12^. The activation barrier for the diffusion of H_*i*_ was determined to be 0.4–0.5 eV, corroborating the occurrence of thermally unstable species as suggested by theoretical calculations^2^,^4^,^11^,^12^. The H_*i*_ and hydrogen trapped within the O vacancy site (H_O_) were believed to be the cause of *n*-type conductivity. The Hydroxyl group on the ZnO surface also exhibits pronounced effects on the chemical activity and electronic properties of oxide surfaces^13^,^14^. The hydroxyl groups on ZnO have been recently identified by observing their vibrational modes by infrared spectroscopy^13^,^14^.

^1^H NMR spectroscopy can be one of the best method to identify hydrogen species and to observe their diffusion properties in ZnO. The dynamical properties of mobile protons in the lattice site of ZnO were previously investigated by ^1^H NMR spectroscopy^15^,^16^,^17^. However, the assignment of interstitial H and surface hydroxyl group on the NMR spectra is a matter of controversy^15^,^16^. Previously, Wang *et al.* attributed the NMR line at 4.8 ppm to the mobile proton in the lattice of ZnO^15^. In contrast, it has been recently reported that the resonance line is due to the hydroxyl group on the surface of ZnO^16^. Thus, the identification of H_*i*_ and surface hydroxyl group on ZnO and their diffusion properties are still not clear.

NMR relaxometry is a powerful technique of atomic-scale access to probe ion hopping motion in solids^18^,^19^,^20^. The laboratory-frame relaxation rate is effective for probing nuclear spin precessing in radio frequency range, i.e., fast diffusing spins. The rotating-frame relaxation rate, on the other hand, effectively probes motions occurring at ultralow-frequencies^21^. Mobile and rigid hydrogen species in ZnO have distinct values for the spectral density in the ultralow-frequency region, thus giving an opportunity to simultaneously investigate their diffusion properties^17^,^21^. Here we identify comprehensive hydrogen species in proton-implanted ZnO nanorods and investigate their dynamical properties by employing the rotating-frame spin-lattice relaxation technique. Our work gives manifest evidence for the first time from a microscopic point of view that implanted protons become mobile in the lattice.

## Results and Discussion

**Figure 1:**
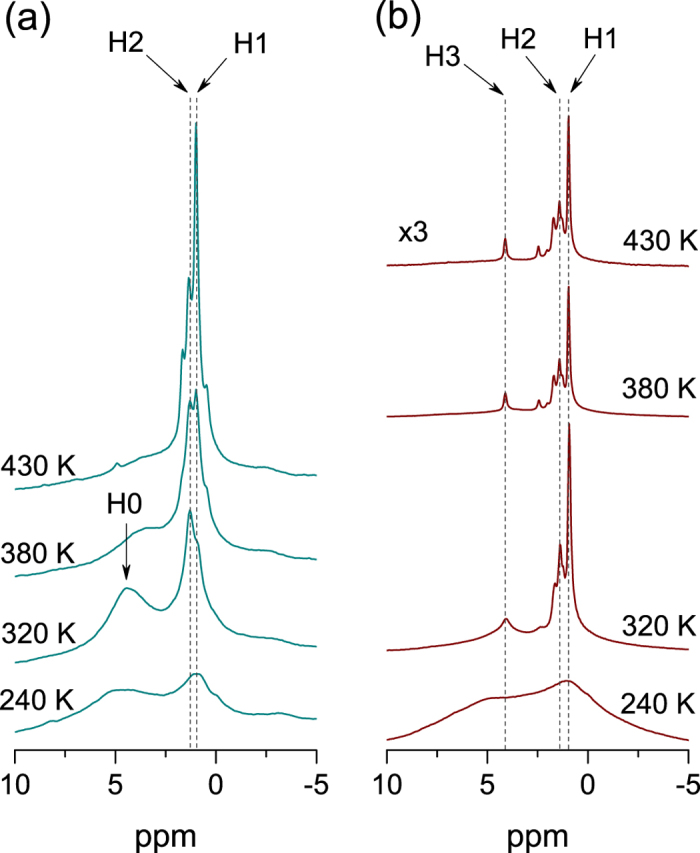
^1^H MAS NMR spectra for the samples (a) before and (b) after irradiation at various temperatures. The H1 and H2 represent the resonance lines at 1.0 and 1.4 ppm before and after irradiation, respectively. A broad resonance line before irradiation is denoted by H0, which is shifted upfield with increasing temperature. The resonance line at 4.1 ppm after irradiation is denoted by H3.

Figure 1 shows the Fourier-transformed ^1^H NMR spectra at various temperatures for ZnO nanorods before and after irradiation. Before irradiation, in Fig. 1(a), motionally narrowed NMR lines with increasing temperature appear at ~1 ppm, as denoted by H1 and H2. The origin of the peaks at ~1 ppm is still not clear, which was previously attributed to the surface OH group, H_O_, or methyl group^15^,^16^,^22^,^23^,^24^,^25^. It is noted that thermal activation barrier of H_O_ in ZnO or the isomerization reaction in methyl group is greater than ~0.7 eV^2^,^4^, whereas that of H atoms from OH group on ZnO surface was reported to be ~0.5 eV^14^,^22^,^25^. After irradiation, in Fig. 1(b), the peak at ~1 ppm separated into three peaks at 1.0 (H1), 1.4 (H2), and 1.7 ppm with increasing temperatures, reflecting slightly different chemical environments around this hydrogen species. Interestingly, narrower lines at 4.1 ppm (H3) with increasing temperature appear up to the highest temperature, indicating obviously distinct hydrogen species with distinct dynamical features compared to the broad line (H0) before irradiation.

**Figure 2:**
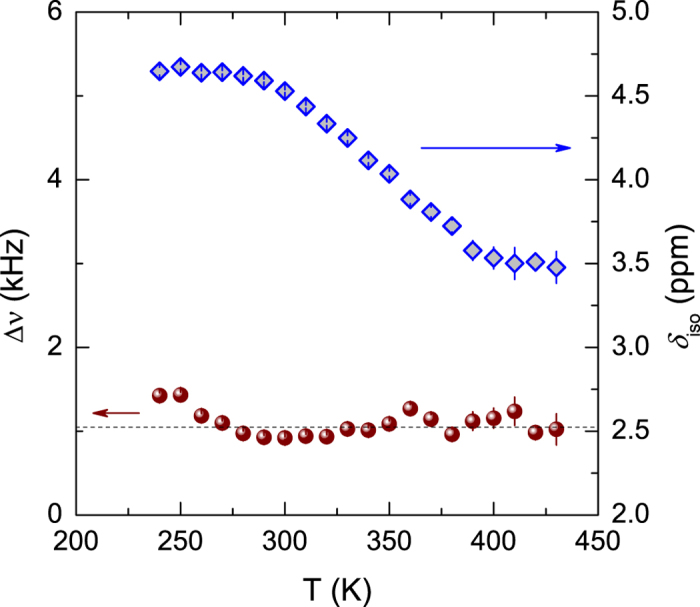
Temperature dependence of the linewidth, Δ*v* (left axis) and isotropic chemical shift, *δ*_iso_ (right axis) of the broad line of H0 before irradiation.

The temperature dependence of the linewidth and chemical shift of H0 is displayed in Fig. 2. The H0 proton exhibits relatively broad lines (Δ*ν* ~1.1 kHz) up to the highest temperature, indicating thermally stable species unlike the other proton species undergoing motional narrowing. Larger ^1^H NMR line shifts (~1 ppm) of H0 to lower ppm values with increasing temperature are shown in the range of around 300 to 400 K (upfield). The chemical shifts of the other protons, on the other hand, are less than 0.3 ppm in all the samples. The greater resonance shifts may be due to a greater change in the bond length and/or strength compared to that of the other resonance lines^26^. Water molecules adsorbed on ZnO surface can form hydrogen bonds showing greater temperature dependence on chemical shift, as well as can be static^27^,^28^. They can be introduced into the lattice during synthesis, exhibiting a broad resonance line due to effective proton-proton dipolar interactions of static single water molecules^16^,^27^. Considering the distinct linewidth changes between H0 and the other proton species as a function of temperature, the chemical exchange between them is unfavourable^29^,^30^. In previous works, a narrow NMR line at ~4.1–4.8 ppm was only observed in the sample synthesized at low temperature, arising from the mobile protons in the lattice site^15^,^16^,^31^. In the present work, however, the motionally narrowed line seems to disappear, indicating thermally unstable proton species diffuses out of the unirradiated sample^15^,^16^,^17^.

**Figure 3:**
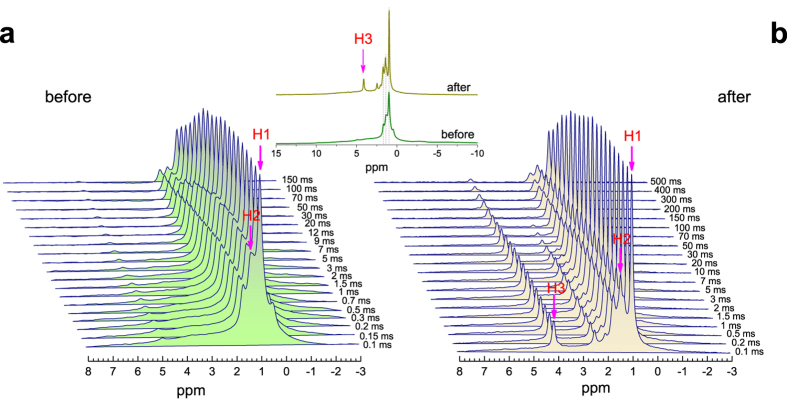
^1^H MAS NMR spectra for the samples (a) before and (b) after irradiation at 430 K following various spin-locking pulse lengths. Vertical arrows denote the peaks at 1.0 (H1) and 1.4 ppm (H2) before irradiation, as well as those and 4.1 ppm (H3) after irradiation. Inset shows the spectra before and after irradiation following a spin-locking pulse of 0.1 ms for comparison.

Figure 3 shows the spectra at 430 K following various spin-locking pulse lengths before and after irradiation. Before irradiation, the spectra exhibit the multiple lines at ~1 ppm with a negligible peak at 4.9 ppm, comparable relaxation times of H1 and H2 being seen from the time-evolution. After irradiation, an apparent narrow NMR line at 4.1 ppm (H3) as well as a smaller peak at 2.4 ppm appear. It is a clear evidence that the narrow line of H3 after irradiation arises from the implanted protons, superimposing the broad one of H0 before irradiation. It is seen from time-evolution that the peaks of H1, H2, and H3 have comparable relaxation times after irradiation. Inset shows the spectra at 430 K before and after irradiation, in which three resonance lines at around 1 ppm after irradiation well correspond to those before irradiation. The concentration of H3 created by the irradiation was estimated to be ~3% of all the hydrogens in the irradiated sample from the intensity of our NMR spectra.

**Figure 4:**
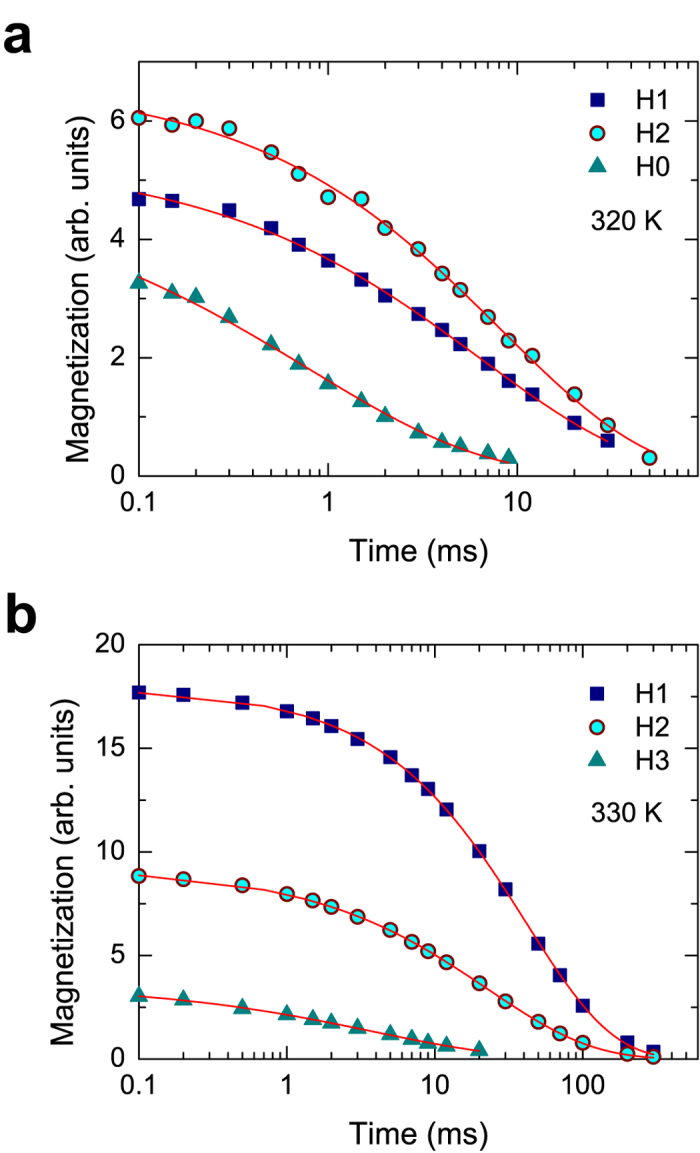
^1^H NMR rotating-frame spin-lattice relaxation patterns. The relaxation patterns shown were measured (**a**) at 320 K before irradiation, and (**b**) at 330 K after irradiation, the solid lines representing fits with stretched exponentials according to Eq. (1). The stretching exponents *β* as a function of temperature are displayed in insets of Figs 5 and 6.

**Figure 5:**
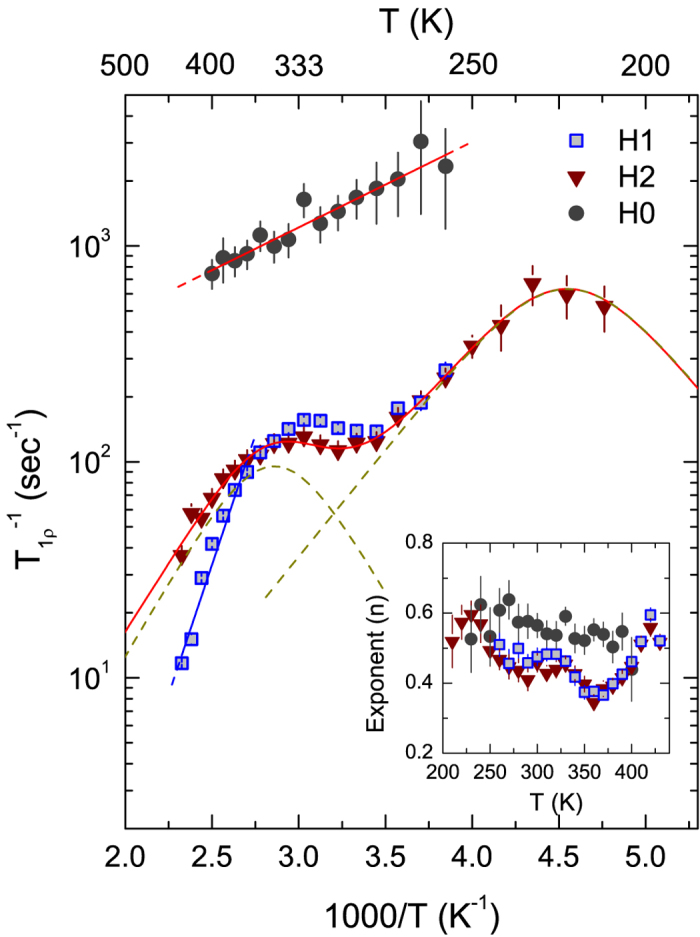
Temperature-dependent ^1^H NMR 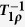 data for the sample before irradiation. The rates of H0 and H1 could not be obtained at lower temperatures, since there is an ambiguity for peak deconvolution for the respective signals. The solid lines of H0 and H1 (high-*T* flank) show fits according to an Arrhenius relation. The solid line of H2 represents a fit with the sum of two independent BPP-type curves, and the dashed lines represent the respective fits to H2. Inset shows the temperature dependence of the best fit values of *n* according to Eq. (1).

**Figure 6:**
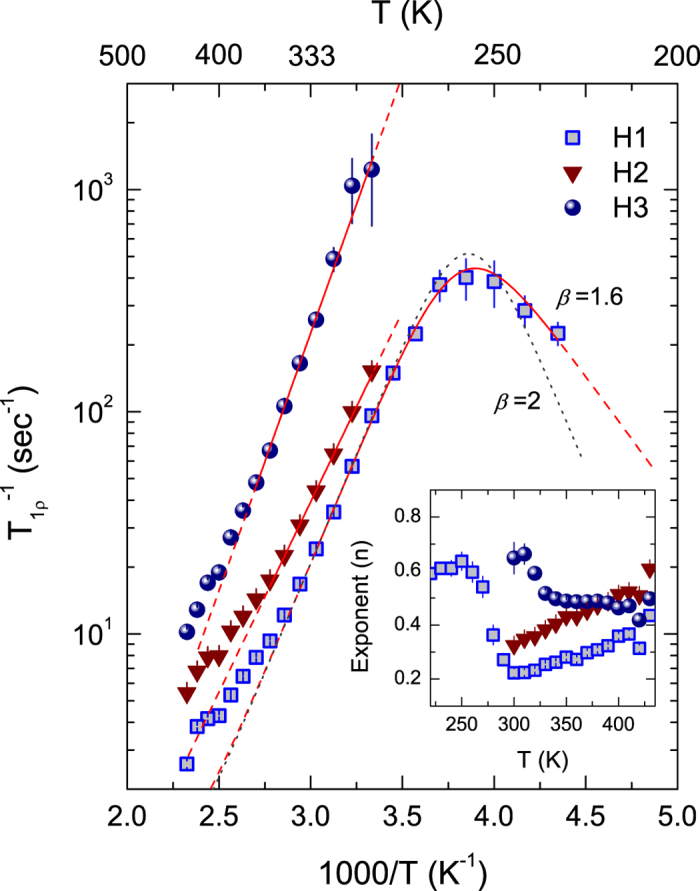
Temperature-dependent ^1^H NMR 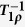 data for the sample after irradiation. The rates of H2 and H3 were obtained above 300 K, since there is an ambiguity for peak deconvolution for the respective signals below 300 K. The solid lines of H2 and H3 show fits according to an Arrhenius relation. The solid line of H1 shows a fit according to Eqs. (2) and (3). The dashed line indicates the deviation of the 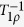 data from simple BPP-type behavior characterized by *β* = 2. Inset shows the temperature dependence of the best fit values of *n* according to Eq. (1).

Figure 4 shows the rotating-frame spin-lattice relaxation patterns corresponding to the signals of H1, H2, and H0 at 320 K before irradiation, and those of H1, H2, and H3 at 330 K after irradiation. The rotating-frame relaxation data were well fitted by a stretched exponential form over the whole temperature range^32^,^33^,


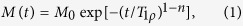


giving a time constant *T*_1*ρ*_ and a stretching exponent *n* for all the three resonance lines. Stretched exponential relaxation can be the result of heterogenous dynamics, reflecting a superposition of different correlation functions for the proton motions^19^,^21^,^32^. Upon increasing *n*, a random distribution of correlation functions increase, leading to an inhomogeneous system^18^,^32^. The stretched exponential behavior for each resonance line was only shown in the present sample, unlike in previous sample synthesized at the temperature as low as 573 K, in which double-exponential type of spin-lattice relaxation was observed^17^. The stretching exponents for each resonance line obtained by the fit according to Eq. (1) are shown as a function of temperature in insets of Figs 5 and 6.

Figure 5 shows the temperature dependency of the rotating-frame spin-lattice relaxation rate (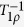) for H1, H2, and H0 before irradiation. H0 decays faster than H1 and H2, and also shows markedly different relaxation behavior from H1 and H2. The activation barrier of H0 was obtained to be *E*_*a*_ = 0.08 eV by a fit according to the Arrhenius relation. Considering the broad NMR lines at the elevated temperatures and small activation energy of H0, we interpret these features as a result from strictly localized motion, corresponding to thermally stable hydrogen species of the water molecules^17^,^27^,^34^,^35^,^36^. Furthermore, the exponent *n* of H0 is not sensitive to temperature, which is valid for the so-called non-diffusive temperature regime (see inset of Fig. 5)^21^. In the case of H1 and H2, on the other hand, similar behavior of the relaxation rates as well as the stretching exponents *n* suggests that they are the same species in different local environments.

The rotating-frame spin-lattice relaxation rate due to the dipolar interaction is given by^19^,^20^


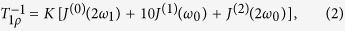


where *K* is the dipole-dipole relaxation constant, and *J*^(*q*)^(*ω*_0(1)_) with *q* = 0, 1, 2 denote spectral densities characterizing magnetic field fluctuations due to three-dimensional ion hopping. The *K* is related with the proton-proton distance according to *K* = 3/10 ⋅ *γ*^4^*ħ*^2^*r*^−6^(*μ*_0_/4*π*)^2^, where *γ* is the gyromagnetic ratio of ^1^H and *r* is the proton-proton distance^17^. A Lorentzian-shaped spectral density function can be described by^19^,^20^,^37^


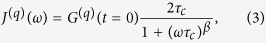


where *G*(*t*) denotes the correlation function assumed to be an exponential, giving the temporal information of the atomic diffusion. The temperature dependence of the correlation time is described by the Arrhenius relation *τ*_*c*_ = *τ*_0_ exp(*E*_*a*_/*k*_*B*_*T*). The original Bloembergen, Purcell, and Pound (BPP) model was developed for uncorrelated jump diffusion with *β* = 2 in Eq. (3), leading to symmetric rate peaks^19^.

In Fig. 5, the H2 data showing two rate maxima were well fitted by the original BPP model with *β* = 2 over the whole temperatures. By the fit according to Eqs. (2) and (3) in the low-temperature regime, the activation barrier and the correlation time at infinite-temperature were thus obtained to be *E*_*a*_ = 0.20 eV and *τ*_0_ = 3.2 × 10^−11^ s, respectively (Table 1). In the high-*T* regime, those were 0.27 eV and 1.3 × 10^−10^ s, respectively. The interproton distances calculated from the fit were *r* = 2.5 and 3.4 Å in the low- and high-*T* regime, respectively. On the high-*T* flank (*ω*_1_*τ*_*c*_ ≪ 1) of H1 data, a fit by the Arrhenius relation gives *E*_*a*_ = 0.48 eV, indicative of more diffusive motion in comparison to that of H2 possibly due to a distinct local environment^2^,^4^. This activation barrier of H1 is comparable to the formation energy (~0.5 eV) of OH species on the nonpolar surfaces^14^. Considering defect sites allowing the adsorption of hydrogen on ZnO, the resonance lines of H1 and H2 may correspond to the surface OH group^14^,^16^. We thus exclude the assignment of these peaks from stable hydrogen species of H_O_, as previously identified by the double resonance technique^15^. The distance *r* characterizes the dipole-dipole distance responsible for the relaxation mechanism based on the BPP theory, longer distance being more likely to be long-range motion^37^. Thus, the shorter *r* and smaller *E*_*a*_ at low-*T* imply short-range motion such as reorientation motion around an oxygen atom, however, in the high-*T* regime long-range motion is quite favorable due to longer *r* and greater *E*_*a*_^2^,^4^,^38^.

Figure 6 shows the temperature dependency of the 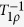 data after irradiation. The fits to the data of H2 and H3 according to the Arrhenius relation give the activation energies of *E*_*a*_ = 0.34 and 0.46 eV, respectively (Table 2). Asymmetric rate peaks of *T*_1*ρ*_ are predicted from the structurally complex or disordered system, from which correlation effects, for instance, Coulomb interactions affect a broad range of short- and long-range motional processes^19^. The best fit to H1 according to the modified BPP relaxation model yields *E*_*a*_ = 0.39 eV and *τ*_0_ = 2.7 × 10^−14^ s together with the value of *β* = 1.6, taking into account its asymmetry and thus introducing the parameter *β*. The fit to H1 with the exponent *β* (1 < *β* ≤ 2) indicates that after irradiation two distinct motional processes disappear due to a broad range of motional processes, exhibiting only one global maximum instead^19^,^21^,^39^,^40^. For H1, the interproton distance *r* is calculated to be 2.5 Å, consistent with that of H2 in the low-*T* regime before irradiation. The *τ*_0_ of H1 after irradiation is about 3 or 4 orders of magnitude shorter than that of H2 before irradiation, indicating higher jump rates of the H1 protons. Besides, more narrowed NMR lines of H1 compared to H2 support the higher mobility of H1 (Figs 1 and 3). The stretching exponents of H1 and H2 exhibit similar behavior above 300 K unlike that of H3, indicative of increasing inhomogeneity with increasing temperature (inset of Fig. 6). It implies that they are identical species in different local environments, as is the case before irradiation.

The 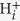 introduced by proton beam irradiation in ZnO causes the resonance line at 4.1 ppm (H3) with its activation barrier of 0.46 eV, corresponding to the hopping barrier of H_*i*_^2^. The resonance line is consistent with the proton in the lattice in terms of the resonance shift and the activation barrier for the sample synthesized at low temperature followed by thermal annealing, thus conclusively ascribed to H_*i*_^2^,^4^,^15^. Furthermore, beam irradiation leads to the correlated jump diffusion of H from the OH group on the surface, thereby yielding a broad range of short- and long-range motion, whereas two distinct motions in different temperature regimes were identified in the unirradiated sample. As a result, our NMR relaxometry reveals that the high-energy beam irradiation with low fluence cannot introduce thermally stable hydrogen with high activation barrier (≥1 eV) in ZnO. We thus explain from a microscopic point of view why protons implanted by high-energy beam irradiation are thermally unstable at room temperature^8^.

## Conclusions

In summary, we have investigated the diffusion properties of hydrogen species in ZnO nanorods before and after high-energy proton beam irradiation. Unlike in unirradiated sample, after irradiation mobile protons at the interstitial site were observed by means of rotating-frame spin-lattice relaxation measurements. The activation energy obtained was 0.46 eV by the Arrhenius relation, corresponding to that of long-range hopping motion. Multiple NMR lines at ~1 ppm, assigned to the hydroxyl group, were observed and their diffusion properties have been investigated before and after irradiation. After irradiation, correlated jump diffusion of this hydroxyl group was observed, indicating a created local structural disorder on the surface. We presented direct evidence in this work that protons introduced by beam irradiation in ZnO occupy a thermally unstable site, as suggested by previous works.

## Methods

ZnO nanorods were synthesized via a sol-gel technique from zinc acetate, (CH_3_COO)_2_Zn·2H_2_O, supplied by Aldrich. To prepare hydrogen doped ZnO, we used a water-soluble linear polymer polyvinyl pyrrolidone following previous works^17^,^41^. Unlike our previous synthesis temperature of 573 K, the samples in this work were synthesized at a higher temperature of 773 K to better remove organic compounds from the ZnO surfaces^17^,^41^. The obtained nanocrystalline powders were then pressed into pellet disks of ~1.5 mm thickness and 10 mm diameter for proton beam irradiation.

The samples were irradiated with 20 MeV proton beams under low fluence condition of 10^12^ cm^−2^, corresponding to beam irradiation time of 600 s, at the Korean Multi-purpose Accelerator Complex. The penetration depth of 20 MeV protons in ZnO were obtained by using the code SRIM (stopping and range of ions in matter). The simulation result shows that most of H^+^ ions can penetrate and stop ~1.2 mm from the top surface^5^,^42^. The ^1^H magic-angle spinning (MAS) NMR measurements were made by using a 400-MHz ^1^H pulsed NMR spectrometer (Bruker Avance II^+^) with a spinning rate of 7 kHz. The rotating-frame spin-lattice relaxation time (*T*_1*ρ*_) data were obtained by applying a 90° pulse. The 90° pulse width used was 3.4 *μ*s, which gave the frequency of the rotating frame *ω*_1_/2*π* = 74 kHz, the *T*_1*ρ*_ data being obtained by varying the length of spin-locking pulse.

## Additional Information

**How to cite this article**: Park, J. K. *et al.* NMR Observation of Mobile Protons in Proton-Implanted ZnO Nanorods. *Sci. Rep.* **6**, 23378; doi: 10.1038/srep23378 (2016).

## Figures and Tables

**Table 1:** Parameters obtained by the fits according to the original BPP relaxation model for H2 and the Arrhenius relation for H1 and H0 of the 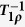 data before irradiation.

	*E*_*a*_ (eV)	*r* (Å)	*τ*_0_ (10^−11^ s)
H1 (high-*T* flank)	0.48(0.03)	–	–
H2 (low-*T*)	0.20(0.03)	2.5(0.1)	3.2(3.9)
H2 (high-*T*)	0.27(0.03)	3.4(0.1)	13(10)
H0	0.08(0.01)	–	–

The numbers in parenthesis denote errors obtained by the fits.

**Table 2:** Parameters obtained by the fits according to the modified BPP relaxation model for H1 and the Arrhenius relation for H2 and H3 of the 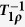 data after irradiation.

	*E*_*a*_ (eV)	*r* (Å)	*τ*_0_ (10^−14^ s)
H1	0.39(0.01)	2.5(0.1)	2.7(0.9)
H2 (high-*T*)	0.34(0.01)	–	–
H3 (high-*T*)	0.46(0.01)	–	–

The fit to the H1 yields an asymmetry parameter *β* = 1.6(±0.2). The numbers in parenthesis denote errors obtained by the fits.
